# Draft Genome Sequence of Entomopathogenic *Serratia liquefaciens* Strain FK01

**DOI:** 10.1128/genomeA.00609-14

**Published:** 2014-06-26

**Authors:** Erika Taira, Kazuhiro Iiyama, Hiroaki Mon, Kazuki Mori, Taiki Akasaka, Kousuke Tashiro, Chisa Yasunaga-Aoki, Jae Man Lee, Takahiro Kusakabe

**Affiliations:** a Laboratory of Silkworm Science, Faculty of Agriculture, Kyushu University, Fukuoka, Japan; b Laboratory of Insect Pathology and Microbial Control, Institute of Biological Control, Faculty of Agriculture, Kyushu University, Fukuoka, Japan; c Laboratory of Molecular Gene Technology, Faculty of Agriculture, Kyushu University, Fukuoka, Japan; d Center for Advanced Instrumental and Educational Supports, Faculty of Agriculture, Kyushu University, Fukuoka, Japan

## Abstract

In the present study, we determined the draft genome sequence of the entomopathogenic bacterium *Serratia liquefaciens* FK01, which is highly virulent to the silkworm. The draft genome is ~5.28 Mb in size, and the G+C content is 55.8%.

## GENOME ANNOUNCEMENT

*Serratia liquefaciens* Kuo1-1, which was originally isolated from the ant lion, is highly virulent to the silkworm, *Bombyx mori*, and the American cockroach, *Periplaneta americana* (1). A spontaneous nalidixic acid-resistant mutant, FK01, was isolated from Kuo1-1 for genetic experiments; this mutant retained virulence against the silkworm (2).

In the present study, a paired-end (fragment size, 400 bp) library of *S. liquefaciens* FK01 was generated. Sequencing was performed using MiSeq sequencing technology with MiSeq Reagent Kits v. 2 (300-cycle kit; Illumina, San Diego, CA, USA). A total of 908,930 reads covering ~136 Mb were yielded after filtering with Trimmomatic v. 0.32 (3). Assembly was performed using Velvet v. 1.2.03 in the DDBJ Read Annotation Pipeline using the following parameters: k-mer, 83; expected coverage, 26; coverage cutoff, 5 (4, 5), resulting in 28 scaffolds. The scaffolds consisted of 49 contigs of 5,279,587 bp. The genome is 5,280,113 bp in length (GC content, 55.8%), with a mean coverage of 25.8-fold. The longest scaffold was 3,043,604 bp, and the *N*_50_ and *N*_90_ sizes of the scaffolds were 3,043,604 bp and 296,035 bp, respectively.

Sequences were annotated using the Microbial Genome Annotation Pipeline (MiGAP) (see http://www.migap.org/) (6). A total of 4,884 protein-coding sequences, 5 rRNAs, and 80 tRNAs were identified in the *S. liquefaciens* FK01 draft genome.

Further analysis of the genome and comparative analyses with related bacteria will contribute to our understanding of the pathogenicity of *S. liquefaciens* in insects.

### Nucleotide sequence accession numbers.

This whole-genome shotgun project has been deposited in DDBJ/ENA/GenBank under the accession number BAZB00000000. The version described in this paper is the first version, BAZB01000000.
